# Pregnenolone sulfate analogues differentially modulate GABA_A_ receptor closed/desensitised states

**DOI:** 10.1111/bph.16143

**Published:** 2023-06-02

**Authors:** Martin Mortensen, Yue Xu, Mohamed A. Shehata, Jacob Krall, Margot Ernst, Bente Frølund, Trevor G. Smart

**Affiliations:** ^1^ Department of Neuroscience, Physiology and Pharmacology University College London London UK; ^2^ Department of Drug Design and Pharmacology, Faculty of Health and Medical Sciences University of Copenhagen Copenhagen Denmark; ^3^ Department of Pathology of the Nervous System, Center for Brain Research Medical University of Vienna Vienna Austria; ^4^ Present address: Xellia Pharmaceuticals ApS Copenhagen Denmark

**Keywords:** chemical analogues, electrophysiology, GABA, GABA_A_ receptor, human embryonic kidney cells, kinetic modelling, molecular dynamics simulations, organic chemistry, pregnenolone sulfate, recombinant expression, synthesis

## Abstract

**Background and Purpose:**

GABA_A_ receptors are regulated by numerous classes of allosteric modulators. However, regulation of receptor macroscopic desensitisation remains largely unexplored and may offer new therapeutic opportunities. Here, we report the emerging potential for modulating desensitisation with analogues of the endogenous inhibitory neurosteroid, pregnenolone sulfate.

**Experimental Approach:**

New pregnenolone sulfate analogues were synthesised incorporating various heterocyclic substitutions located at the C‐21 position on ring D. The pharmacological profiles of these compounds were assessed using electrophysiology and recombinant GABA_A_ receptors together with mutagenesis, molecular dynamics simulations, structural modelling and kinetic simulations.

**Key Results:**

All seven analogues retained a negative allosteric modulatory capability whilst exhibiting diverse potencies. Interestingly, we observed differential effects on GABA current decay by compounds incorporating either a six‐ (compound **5**) or five‐membered heterocyclic ring (compound **6**) on C‐21, which was independent of their potencies as inhibitors. We propose that differences in molecular charges, and the targeted binding of analogues to specific states of the GABA_A_ receptor, are the most likely cause of the distinctive functional profiles.

**Conclusions and Implications:**

Our findings reveal that heterocyclic addition to inhibitory neurosteroids not only affected their potency and macroscopic efficacy but also affected innate receptor mechanisms that underlie desensitisation. Acute modulation of macroscopic desensitisation will determine the degree and duration of GABA inhibition, which are vital for the integration of neural circuit activity. Discovery of this form of modulation could present an opportunity for next‐generation GABA_A_ receptor drug design and development.

## Graphical abstract

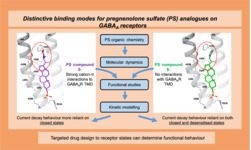

**Abbreviations**
DHEAS: dehydro‐epiandrosterone sulfate
ESI: electrospray ionisation source
HR‐MS: high‐resolution mass spectroscopy
MD: molecular dynamics
PS: pregnenolone sulfate
THDOC: tetrahydro‐deoxycorticosterone sulfate
TMD: transmembrane domain
TNCG: Truncated Newton Conjugate Gradient
τ_w_
: weighted time constant

**What is already known**

- Sulfated neurosteroids in the brain act as antagonists at GABA_A_Rs.
- These inhibitory neurosteroids bind within the transmembrane domain of the receptor.

**What does this study add**

- New neurosteroids are presented based on pregnenolone sulfate with new heterocyclic substituents on ring D.
- These new pregnenolone sulfate analogues show binding preference to particular states of the GABA_A_R.

**What is the clinical significance**

- These analogues may form the basis for therapeutics targeting GABA_A_R states and treating neurological disease.

## 1 INTRODUCTION

γ‐Aminobutyric acid (GABA) is a major neurotransmitter in the central nervous system (CNS), with an inhibitory role mediated by the activation of GABA_A_ receptors (GABA_A_Rs). Because of their involvement in a plethora of neurophysiological and pathophysiological processes, modulation of GABA_A_Rs holds considerable therapeutic potential and promise (Mohler, 2012; Sieghart & Saviç, 2018; Smart, 2015).

**FIGURE 1:**
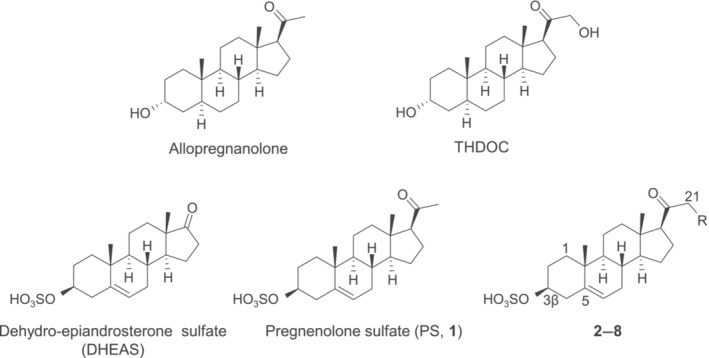
Structures for the potentiating neurosteroids allopregnanolone and tetrahydro‐deoxycorticosterone (THDOC) and the sulfated inhibitory steroids dehydro‐epiandrosterone sulfate (DHEAS), pregnenolone sulfate (PS; designated as compound **1**) and the novel base (lead) structure for the PS analogues synthesised in this study (yielding compounds **2**–**8**, where R = Br or an *N*‐heterocycle).

These receptors are heteropentamers belonging to the superfamily of ligand (neurotransmitter)‐gated ion channels (Barnard et al., 1987). Subunits are concentrically arranged to form a central pore selectively permeable to mainly Cl^−^ and to HCO_3_^−^ ions (Ernst et al., 2005; Farrant & Kaila, 2007). The entire membrane‐spanning structure is composed of an extracellular domain (ECD), a transmembrane domain (TMD) and an intracellular domain (ICD), most of which, with the exception of the ICD, is now resolved at high resolution following structural studies using X‐ray crystallography and cryo‐electron microscopy (Laverty et al., 2019; Miller & Smart, 2010; Phulera et al., 2018; Zhu et al., 2018). Being an allosteric receptor complex, GABA‐evoked signalling can be modulated by ligands targeting binding sites located in several domains (Puthenkalam et al., 2016). One of the most potent groups of endogenous modulators is the neurosteroids (NS), which exhibit a range of effects from potentiation of GABA responses and direct receptor activation (tetrahydrodeoxycorticosterone [THDOC] and allopregnanolone), to inhibition. The latter property is exemplified by naturally occurring pregnenolone sulfate (PS) and dehydroepiandrosterone sulfate (DHEAS) (Belelli & Lambert, 2005; Seljeset et al., 2015). For NS structures, see Figure 1.

The physiological importance of endogenous NS modulation of GABA_A_Rs is of significant interest, and at least two sites of action have been revealed from structural and photo‐labelling studies (Chen et al., 2019; Laverty et al., 2017; Miller et al., 2017; Sugasawa et al., 2020). Whereas potentiating NS bind across the TMD interface formed by principal β and complementary α subunits at the lipid interface, the inhibitory NS seemingly bind to a discrete intra‐subunit TMD site involving M3 and M4, which offers a basis for molecular interpretation of NS action on GABA_A_R function (Laverty et al., 2017; Miller et al., 2017). Although there may be other sites for binding of inhibitory NS, this (TMD) is an interesting location from a functional perspective, because it brings the inhibitory NS into proximity with the receptor's desensitisation gate located at the base of the ion channel (Gielen et al., 2015; Laverty et al., 2017, 2019). Moreover, structure–function studies in the vicinity of the desensitisation gate, using recombinant αβγ GABA_A_Rs, also indicated that the TMD, and in particular M3 and M4 of the α subunit facing the membrane lipid phase, was important for the binding and subsequent inhibitory activity of PS (Seljeset et al., 2018). Rapid application of GABA to recombinant αβγ receptors also revealed marked inhibition of the steady‐state current compared with the peak GABA current, in accord with enhanced macroscopic desensitisation forming the major mode of PS inhibition (Seljeset et al., 2018).

Although structure–activity studies based on naturally occurring and synthetic inhibitory NS have been reported (Akk et al., 2007; Sugasawa et al., 2020), the chemical determinants that mediate NS‐induced inhibition at GABA_A_Rs are still not yet fully explored or understood (Baker et al., 2010; Seljeset et al., 2015). Inhibitory NS commonly feature a signature sulfate group in the C‐3β position and a double bond in ring B (C5–C6), whilst also demonstrating considerable chemical tolerance in terms of the structure and conformation that preserves the inhibitory effect (Park‐Chung et al., 1999; Wang et al., 2002).

Previously, the C‐21 position (on ring D) of pregnanolone has been extensively explored in conjunction with positive allosteric modulation by NS, where the addition of specific heterocycles at this location on the pregnanolone ring scaffold is broadly tolerated, affording analogues with variable potency at synaptic‐ and extrasynaptic‐type GABA_A_Rs (Martinez et al., 2015; Martinez et al., 2017). In comparison, the effects of corresponding C‐21‐based structural changes on the functional profiles of inhibitory NS are relatively unexplored. In this study, we probe the importance of C‐21 in PS for inhibitory allosteric modulation by NS. This study required the synthesis of a series of de novo C‐21 *N*‐heterocycle‐substituted analogues that were evaluated for their functional profiles using electrophysiology, molecular dynamics (MD) simulations, site‐directed mutagenesis, and structural and kinetic modelling studies.

These experimental approaches have provided new insight into the mechanism of action of inhibitory PS‐related NS. Specifically, our PS analogues caused differential effects on GABA_A_R macroscopic desensitisation potentially linked to the stabilisation of receptor conformation in specific closed/preactivated/desensitised states. We propose that this mechanism can become a new feature of GABA_A_R modulation, which may be exploited for future drug development.

## 2 METHODS

### 2.1 Organic chemistry

All reagents and solvents were obtained from commercial suppliers and used without further purification. Thin‐layer chromatography (TLC) was carried out using Merck silica gel 60 F254 plates, and reactions were monitored with potassium permanganate (KMnO_4_) spray reagent. Flash column chromatography for purification was achieved by Merck silica gel (0.040–0.063 mm). Analytical high‐performance liquid chromatography (HPLC) was carried out on a Thermos Scientific Dionex 3000 Ultimate instrument connected to a Gemini‐NX 3u C18 110A (250 × 4.6 mm) column. The purity of all the final compounds was determined by analytic HPLC with gradient mobile phase (A: H_2_O/TFA = 100/0.1; B: acetonitrile/H_2_O/TFA = 90/10/0.1 30%B–100%B in 20 min), achieving >95%. Preparative HPLC was performed on a Thermos Scientific Dionex 3000 Ultimate instrument with a Gemini‐NX 5u RP C18 column (250 × 21.2 mm) using the solvent A (H_2_O 100%) and solvent B (MeCN/H_2_O = 90/10) with the same gradient programme as analytic HPLC. Accurate mass of the final compounds was detected by ultra‐performance liquid chromatography‐mass spectrometry (UPLC‐MS) with Water Acquity UPLC and electrospray ionisation source (ESI) or HPLC‐high‐resolution mass spectroscopy (HR‐MS). Detailed chemistry conditions used in the synthesis of compounds **2**–**8** are included in the supporting information.

### 2.2 MD methodology

Prior to initiating MD simulations, we performed a pKa analysis using the Jaguar pKa prediction tool in Maestro Schrödinger (Bochevarov et al., 2016; Schrödinger Release 2018‐1: Maestro, Schrödinger, LLC, NY, 2018) to predict ionisation states for each ligand. The PS analogues were then manually superimposed on the PS‐bound GABA_A_R chimera crystal structure (Laverty et al., 2017) (PDB 5OSC). The ligand–protein complex was energy minimised using MacroModel Schrödinger (Schrödinger Release 2018‐1). The Truncated Newton Conjugate Gradient (TNCG) minimisation method was selected with maximum iteration steps set to 5000 and the convergence gradient to 0.001. The MD simulations were performed using the Desmond package in Maestro Schrödinger. Simulations were run for 12 ns on each ligand–receptor complex in the predefined POPC membrane model. Explicit water molecules were handled using the simple point charge model. Constant temperature and pressure were applied at 300 K and 1.01325 bar, respectively. The system was coupled to an isotropic Berendsen thermostat and barostat, with relaxation time set to 1 and 2 ps, respectively.

### 2.3 HEK cell culture, mutagenesis and transfections

HEK 293 cells (ATCC Cat# CRL‐1573, RRID:CVCL_0045) were maintained and transfected under standard conditions. In brief, cells were grown in monolayers in Dulbecco's modified Eagle's medium supplemented with 10% v/v fetal calf serum, 100 U·ml^−1^ of penicillin‐G and 100 μg·ml^−1^ of streptomycin and incubated at 37°C in humidified 95% air/5% CO_2_.

Point mutations were introduced into murine GABA_A_R subunit pRK5‐driven cDNAs by standard PCR mutagenesis techniques and confirmed in full‐length DNA sequencing. Before transfection, HEK cells were plated onto 22 mm of glass coverslips (VWR) coated in poly‐l‐lysine (Sigma). Cells were transfected with α1 (wild‐type [wt] or mutant [mut]), β3 (wt), γ2L (wt or mut) and enhanced green fluorescent protein (eGFP) in an equimolar ratio (1:1:1:1), using a calcium‐phosphate precipitation protocol. The transfection solution was prepared from 4 μg of cDNA per coverslip, 20 μl of 340 mM of CaCl_2_ and 24 μl of 2× HEPES‐buffered saline (HBS) (50 mM of HEPES, 280 mM of NaCl and 2.8 mM of Na_2_HPO_4_). Cells were selected for electrophysiology after 16–30 h based on their morphology and moderate GFP fluorescence.

### 2.4 Electrophysiology

Coverslips with transfected HEK cells were transferred into a recording chamber on a Nikon Eclipse FN1 microscope with a 470‐nm LED for GFP fluorescence. Cells were continuously perfused with Krebs solution containing (mM) 140 NaCl, 4.7 KCl, 1.2 MgCl_2_, 2.52 CaCl_2_, 11 glucose and 5 HEPES (pH 7.4). Patch pipettes (thin‐walled filamented borosilicate glass capillaries; TW150F‐4; WPI, USA; 3–4 MΩ) were filled with an intracellular solution containing (mM) 140 CsCl, 2 NaCl, 2 MgCl_2_, 5 EGTA, 10 HEPES, 0.5 CaCl_2_, 2 Na‐ATP and 0.5 Na‐GTP (pH 7.2). Drugs were applied to cells using a Y‐tube delivery system (Mortensen & Smart, 2007), with the PS analogues pre‐applied 10–12 s before co‐application with GABA.

HEK cells were voltage clamped at −30 mV using an Axopatch 200B amplifier (Molecular Devices, USA), and whole‐cell currents were filtered at 5 kHz (−36 dB), digitised at 50 kHz via a Digidata 1322A (Molecular Devices) and recorded to a Dell Optiplex 990 using Clampex 10.2 (Molecular Devices). Series resistance was compensated at 60%–70%, and only data with <20% deviation in series resistance would be included in subsequent analyses.

Weighted time constants for the decay phase (τ_w_) were calculated by fitting a biexponential curve to the current decay waveform and applying the following equation:

$$\tau_{w}=\frac{A_{1}.\tau_{1}+A_{2}.\tau_{2}}{A_{1}+A_{2}},$$

where τ_1_ and τ_2_ represent time constants for the two exponential components of the decay phase and A_1_ and A_2_ are their relative area contributions.

### 2.5 Materials

The PS analogues (compounds **2**–**8**) were prepared with the highest stock concentrations of 100 mM in dimethyl sulfoxide (DMSO). These stocks were diluted at least 1000‐fold, so the maximum final PS analogue concentration achieved was 100 μM. GABA was purchased from Sigma‐Aldrich. All salts and reagents were obtained from Sigma unless indicated otherwise.

### 2.6 Data and statistical analysis

The data and statistical analysis comply with the recommendations of the *British Journal of Pharmacology* on experimental design and analysis in pharmacology (Curtis et al., 2022). For statistical analysis, data fitting and presentation, Prism (Version 9, GraphPad Software, San Diego, USA) and Origin (Version 6 and OriginPro 2020b, OriginLab Corporation, USA) were used.

Current responses from voltage‐clamp experiments were measured, and decays fitted using ClampFit 10.2 (Molecular Devices) to determine the respective time constants. In Origin, data were plotted as concentration–response curves, and curves fitted using the following inhibition equation:

$$I/I_{\max}=1-\left({\left[B\right]}^{n}/\left({{\mathrm{IC}}_{50}}^{n}+{\left[B\right]}^{n}\right)\right),$$

where I is the current response, B is the concentration of the PS analogue, n is the slope coefficient and IC_50_ is the analogue concentration producing 50% inhibition of GABA current (inhibitory potency). Potency values are presented as pIC_50_ with SEM values. The mean was transformed into a molar concentration by pIC_50_ = −log IC_50_. Other data are represented as the mean ± SEM.

Normally distributed datasets are compared using unpaired *t* tests, paired *t* tests and analysis of variance (ANOVA) with Tukey's post hoc tests where appropriate. Statistical significance was defined by *P* < 0.05. No statistical methods were used to predetermine sample size in this study. The experiments were not randomised and the investigators were not blinded during experiments and outcome assessment.

### 2.7 Kinetic modelling and GABA current simulations

We used ChanneLab (Version 2.2; Synaptosoft) to build relatively simple kinetic models of the GABA_A_R. To explore the functional behaviour of PS analogues (compounds) **5** and **6**, we incorporated concentration‐dependent PS analogue‐bound states into the model. Simulated currents were generated in ChanneLab and explored at different GABA and PS analogue concentrations. These were evaluated against GABA currents generated under experimental conditions, using the waveform fitting function in ChanneLab. Rate constants connecting receptor states were determined initially empirically with reference to previously published values and varied individually to optimise the fit to the data (seed values) prior to using waveform fitting and Runge–Kutta numerical integration (RK5) to eventually simulate the experimental GABA current profiles with 50 iterations. GABA‐activated membrane currents were fitted over their full timecourse of 20 s.

### 2.8 Nomenclature of targets and ligands

Key protein targets and ligands in this article are hyperlinked to corresponding entries in http://www.guidetopharmacology.org and are permanently archived in the Concise Guide to PHARMACOLOGY 2021/22: Ion Channels (Alexander et al., 2021).

## 3 RESULTS

In this study, we synthesised seven new analogues of PS. The pharmacological profiles of these compounds were initially assessed using whole‐cell voltage‐clamp electrophysiology with recombinant α1β3γ2L GABA_A_Rs expressed in HEK cells. A subset of analogues with differential effects on the GABA current decay phase were selected for further study. The aim was to explore the underlying mechanisms, how the analogues interact with the TMD‐NS binding site and how this might affect the conformational states of the GABA_A_R.

### 3.1 Chemistry

**FIGURE 2:**
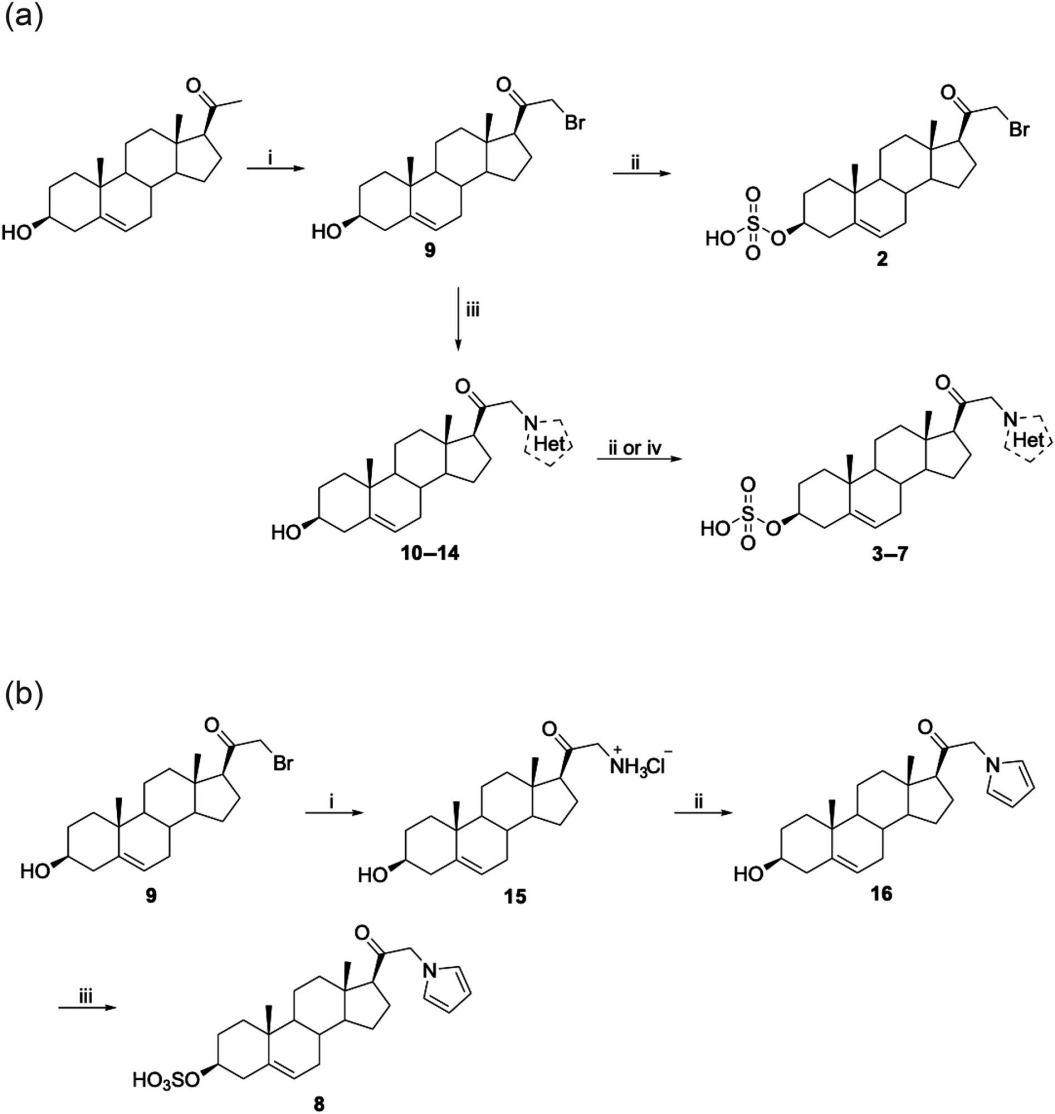
Synthesis of C‐21 pregnenolone sulfate analogues **2**, **3**–**7** (a) and **8** (b). Reagents and conditions: (a), (i) CuBr_2_, pyridine, methanol, reflux; (ii) pyridine sulfur trioxide, CHCl_3_, room temperature (rt); (iii) *N*‐heterocycle, K_2_CO_3_, KI, acetone, reflux; (iv) pyridine sulfur trioxide, DMF, 60°C. (b), (i) hexamethylenetetramine, CHCl_3_ at rt, then HCl, EtOH; (ii) CH_3_COONa, CH_3_COOH, 2,5‐dimethoxytetrahydrofuran, H_2_O, 100°C; (iii) pyridine sulfur trioxide, CHCl_3_, rt.

The synthesised PS analogues (compounds **2**–**8**) were designed to introduce structural diversity into ring D at C‐21 with *N*‐heterocyclic substituents varying in several properties of volume, conformation, aromaticity and ionisation. These compounds were synthesised (Figure 2) with compound **9** being a key intermediate to explore the structure–activity relationship (SAR) at the C‐21 position through nucleophilic *N*‐alkylation with a series of heterocycles. The corresponding C‐3 sulfates were prepared to yield compounds **2** and **3**–**7** (Figure 2a). The C‐21‐pyrrole intermediate **16** was achieved by condensation between the primary amine **15** and 2,5‐dimethoxytetrahydrofuran in the presence of an acid catalyst, generally known as the Clauson–Kaas reaction (Gourlay et al., 2006) before subsequent transformation into the sulfate analogue **8** (Figure 2b).

### 3.2 Pharmacological evaluation

To assess the pharmacological profiles of the PS analogues, we investigated their effects on a common isoform of the GABA_A_R, α1β3γ2L, which is an archetypal synaptic receptor involved in phasic inhibition (Farrant & Nusser, 2005), but also found extrasynaptically where it can contribute to tonic inhibition (Thomas et al., 2005). GABA_A_Rs were expressed in HEK cells and studied using whole‐cell recording combined with fast drug applications to assess the PS analogues. Because PS is a negative allosteric modulator, we studied their effects on receptor activation caused by relatively high (30 μM) GABA concentrations equivalent to ~EC_90_ for α1β2/3γ2L (Mortensen et al., 2011).

**FIGURE 3:**
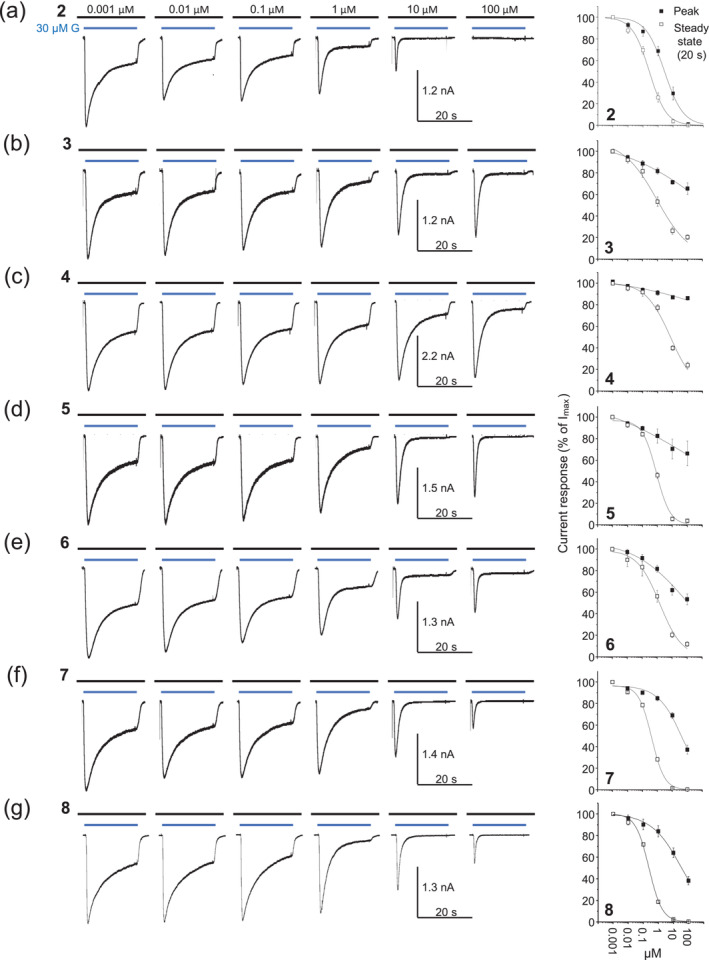
Pregnenolone sulfate (PS) analogues display differential negative allosteric modulatory effects on GABA induced α1β3γ2L GABA_A_R currents. (a–g) Representative current responses to 30 μM of GABA in the presence of a range of PS analogue (**2**–**8**) concentrations that are pre‐applied (for 10–12 s) and co‐applied: **2** (a), **3** (b), **4** (c), **5** (d), **6** (e), **7** (f) and **8** (g) for 20 s. The right column shows the concentration–response relationships for the seven PS analogues measured at the peak (solid squares) and after 20 s (open squares; near steady state) of the GABA‐activated currents. All points are means ± SEM (see Table 1 for n numbers) and are plotted as percentages of the currents evoked by 30 μM of GABA in the absence of a PS analogue.

**FIGURE 4:**
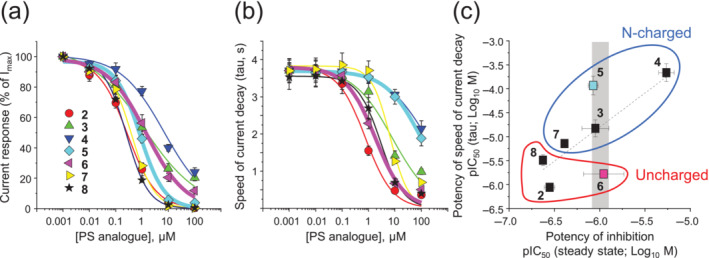
Pregnenolone sulfate (PS) concentration inhibition relationships for steady‐state GABA currents and for the time constant for GABA current decay for α1β3γ2L GABA_A_Rs. (a) Concentration–inhibition relationships for the PS analogues causing steady‐state inhibition of the GABA current. The curves were generated by a modified Hill equation for each PS analogue: **2** (red circle), **3** (green triangle), **4** (dark blue triangle), **5** (light blue diamond), **6** (pink triangle), **7** (yellow triangle) and **8** (black star). (b) Relationship between the decay time for GABA current (weighted tau) plotted against PS analogue concentration. Same colour code as in panel (a). (c) Correlation plot of the pIC_50_ values obtained from the GABA current steady‐state inhibition and exponential GABA current weighted decay time constant (*P* = 0.065, *R* ^2^ = 0.5253). Data are fitted using linear regression (y = 1.423x + 3.738). The grey bar at 1 μM highlights compounds, **3**, **5** and **6**, with near identical potencies for steady‐state current inhibition. N‐charged (blue) and uncharged (red) compounds are grouped. ANOVA of steady‐state IC_50_ potency values for **3**, **5** and **6** revealed no significant difference, whereas for the weighted current decay time constant, *P* < 0.05. The curves (panels a and b) and the data points (panel c) are emphasised for compounds **5** (cyan) and **6** (pink). Data points represent mean values ± SEM (see Table 1 for n numbers).

Endogenous NS are distributed throughout the brain parenchyma enabling long‐term effects on synaptic and extrasynaptic GABA_A_Rs (Reddy, 2010). We therefore used 20 s of GABA applications to investigate their effects on both peak and (near) steady‐state GABA currents. All the PS analogues retained negative allosteric effects with greater potency on steady‐state compared with peak GABA currents (Figure 3 and Table 1). The rank order of peak GABA current inhibition in terms of potency determined by the IC_50_ (μM in parentheses) was compound **2** (2.7) > **8** (33) > **7** (47) > **6** (220) > **3** (743) > **5** (~2000) > **4** (>2000). Compound **2** was clearly the most potent inhibitor of peak current (*P* < 0.05; Figure 3 and Table 1), with compounds **3**–**5** showing relatively little peak current inhibition. The steady‐state GABA current inhibition displayed less variation in potency between the PS analogues, providing a rank order (IC_50_; μM) of compound **8** (0.24) = **2** (0.28) > **7** (0.41) > **5** (0.84) = **3** (0.89) = **6** (1.1) > **4** (5.4) (*P* < 0.05; Figures 3 and 4a and Table 1). Notably, compounds **2** and **8** were the most potent, and compound **4** was again the least potent. Given the ambient paracrine nature of NS levels in the brain, physiologically, steady‐state inhibition was considered the more relevant parameter, and this formed the focus for the study.

**TABLE 1:** C‐21 substituents and inhibition potencies for PS analogues **2**–**8**.

Compound	R	Peak inhibition	Steady‐state inhibition	Decay inhibition
		(pIC_50_; IC_50_)	(pIC_50_; IC_50_)	(pIC_50_; IC_50_)
**2**	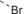	5.577 ± 0.140 (2.7 μM; n = 8)	6.555 ± 0.058 (0.28 μM; n = 8)	6.058 ± 0.078 (0.88 μM; n = 7)
**3**	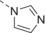	3.129 ± 0.688 (743 μM; n = 6)	6.050 ± 0.149 (0.89 μM; n = 6)	5.070 ± 0.170 (8.5 μM; n = 5)
**4**	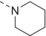	n.m. (>2 mM; n = 6)	5.265 ± 0.085 (5.4 μM; n = 6)	3.663 ± 0.186 (217 μM; n = 6)
**5**	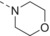	est. (~2.7) (~2 mM; n = 6)	6.074 ± 0.054 (0.84 μM; n = 6)	3.933 ± 0.194 (117 μM; n = 6)
**6**	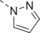	3.658 ± 0.243 (220 μM; n = 6)	5.946 ± 0.188 (1.1 μM; n = 6)	5.781 ± 0.047 (1.7 μM; n = 7)
**7**	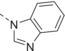	4.327 ± 0.128 (47 μM; n = 6)	6.391 ± 0.038 (0.41 μM; n = 6)	5.142 ± 0.087 (7.2 μM; n = 6)
**8**	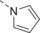	4.478 ± 0.128 (33 μM; n = 6)	6.627 ± 0.025 (0.24 μM; n = 6)	5.491 ± 0.115 (3.2 μM; n = 6)

*Note*: Table 1 shows pIC_50_ and IC_50_ values for peak, steady‐state and GABA current decay for α1β3γ2L for each of the pregnenolone sulfate (PS) analogues (compounds) identified by their different *N*‐heterocyclic substituents. IC_50_ values are shown as mean ± SEM, where n is the number of experiments. If peak inhibition curves only provided partial information for sigmoidal fits, an IC_50_ value was estimated by graphical extrapolation and noted as ‘est.’; but where sigmoidal fits could not be generated, the IC_50_ value was designated ‘not measurable’ (n.m.).

After assessing inhibitory potency, we then examined the GABA current decay times for which the PS analogues had differential effects. At the lowest concentration of each PS analogue (1 nM), the mean (weighted) decay times (τ_w_) were comparable at ~3.6–3.8 s for compounds **2**–**8** (**2**: 3.8 ± 0.2 s; **3**: 3.8 ± 0.8; **4**: 3.7 ± 0.3; **5**: 3.7 ± 0.1; **6**: 3.8 ± 0.2; **7**: 3.8 ± 0.2; and **8**: 3.6 ± 0.3; Figure 4b), which is similar to the speed of desensitisation for α1β3γ2L receptors in the presence of a GABA‐EC_90_. Increasing the PS analogue concentration caused faster current decays that will reflect an increased inhibition, and increased macroscopic desensitisation, but which may also reflect changes to other receptor states including those involved in transitioning of GABA‐bound inactive to GABA‐bound receptor activated states (i.e., preactivation states). Thus, the macroscopic current decay alone is not a precise guide to underlying changes in microscopic desensitisation states (Bianchi et al., 2007). The decay speed increased with the PS analogue concentration yielding a rank potency order (based on IC_50_ values) of compound **2** (0.88 μM) > **6** (1.7) > **8** (3.2) > **7** (7.2) = **3** (8.4) > **5** (117) > **4** (217) (*P* < 0.05) with compounds **4** and **5** exhibiting the lowest and compounds **2**, **6** and **8** the highest potency effects on GABA current decay speeds (Figure 4b and Table 1).

Normally, we would expect a potent inhibitor, which is dependent on receptor activation for binding and subsequent inhibition, to result in fast current decay times, whereas lower potency antagonists would lead to slower decay times. We examined the inhibition profiles of the PS analogues by plotting the potency of steady‐state GABA current inhibition against the potency of increasing GABA current decay speed for each PS analogue. This revealed a correlative tendency between these parameters and an unexpected scatter around the linear regression line (Figure 4c). Compounds **3**, **5** and **6** were distinctive possessing near identical potencies for steady‐state current inhibition at ~1 μM, coupled to a 2‐log range over which the potencies varied for their corresponding effects on GABA current decay speed (Figure 4c and Table 1). Assuming the speed of receptor macroscopic desensitisation is initially constant and defined by the receptor isoform, we deduced that these differential potency effects on current decay are linked to the speed of inhibition induced by the PS analogues.

Comparing the C‐21 substituents on the three PS analogues revealed that compounds **3** and **6** had high heterocycle ring shape similarity (five‐membered imidazole vs. pyrazole, respectively); however, the 70‐fold difference in current decay observed between compound **5** (six‐membered morpholine) and compound **6** (pyrazole) was notable. Any alterations to 3D shape and volume, charge disposition and polarity centred on C‐21 will likely impact on the binding interactions within the PS site. Such differences could cause distinct conformational changes to the receptor protein during activation, ultimately affecting current decay kinetics. To investigate the chemical characteristics of the PS analogues **3**, **5** and **6**, we used a predictive pKa analysis to investigate potential differences in charge at neutral pH in aqueous solution followed by MD simulations.

### 3.3 Computational modelling

We computationally predicted the basicity (tendency to act as a proton acceptor) of nitrogen atoms possessing lone pairs of electrons in the heterocycles of compounds **3**, **5** and **6** (Table 2). Although, in our electrophysiological assays, PS analogues were dissolved in a neutral aqueous salt solution, on applying to GABA_A_Rs, these ligands would transfer from the aqueous phase to accumulate in the hydrophobic phospholipid cell membrane before accessing their protein (TMD) binding site (Akk et al., 2009). Consequently, DMSO was chosen as the solvent when calculating all pKa values for nitrogen atoms with lone pair electrons in PS analogues **3**, **5** and **6**, because both DMSO and the lipid membrane can be treated as aprotic solvents (i.e., no hydrogen atoms present on nitrogen or oxygen atoms). This analysis revealed a clear difference between the predicted heterocycle pKa values. Compounds **3** and **5** possessed a positively charged nitrogen atom under the conditions of our pharmacological characterisation, whereas compound **6** was predicted to be neutral (Table 2). Because compound **6** was more potent at promoting GABA current decay, this could suggest that **6** has a different binding mode from compounds **3** and **5** at this PS binding site, resulting in this differential high potency effect. This was investigated further using MD simulations for PS analogues **3**, **5** and **6**.

**TABLE 2:** Analysis of charge in PS analogues.

Compound	pKa (DMSO)	N‐charge	Structure in MD
**3**	9.9	+1	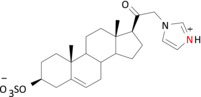
**5**	7.6	+1	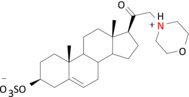
**6**	4.8	0	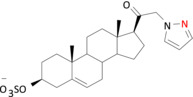

*Note*: Table 2 shows in silico calculated pKa values for the nitrogen atoms with lone pair electrons for pregnenolone sulfate (PS) analogues **3**, **5** and **6**. The nitrogen atoms are labelled in red and the predicted charges are shown.

Abbreviations: DMSO, dimethyl sulfoxide; MD, molecular dynamics.

Each compound was superimposed on the PS‐bound GABA_A_R crystal structure (PDB 5OSC) as a starting template to explore their binding poses within the TMD of the α1 subunit. Protein–ligand complexes were energy minimised to ensure no unfavourable contacts occurred between each analogue and residue side chains in the binding site. A 12‐ns MD simulation revealed a clear distinction between the binding site interaction patterns for positively charged analogues **3** and **5** (Movies S1 and S2) compared with the neutral compound **6** (Movie S3).

**FIGURE 5:**
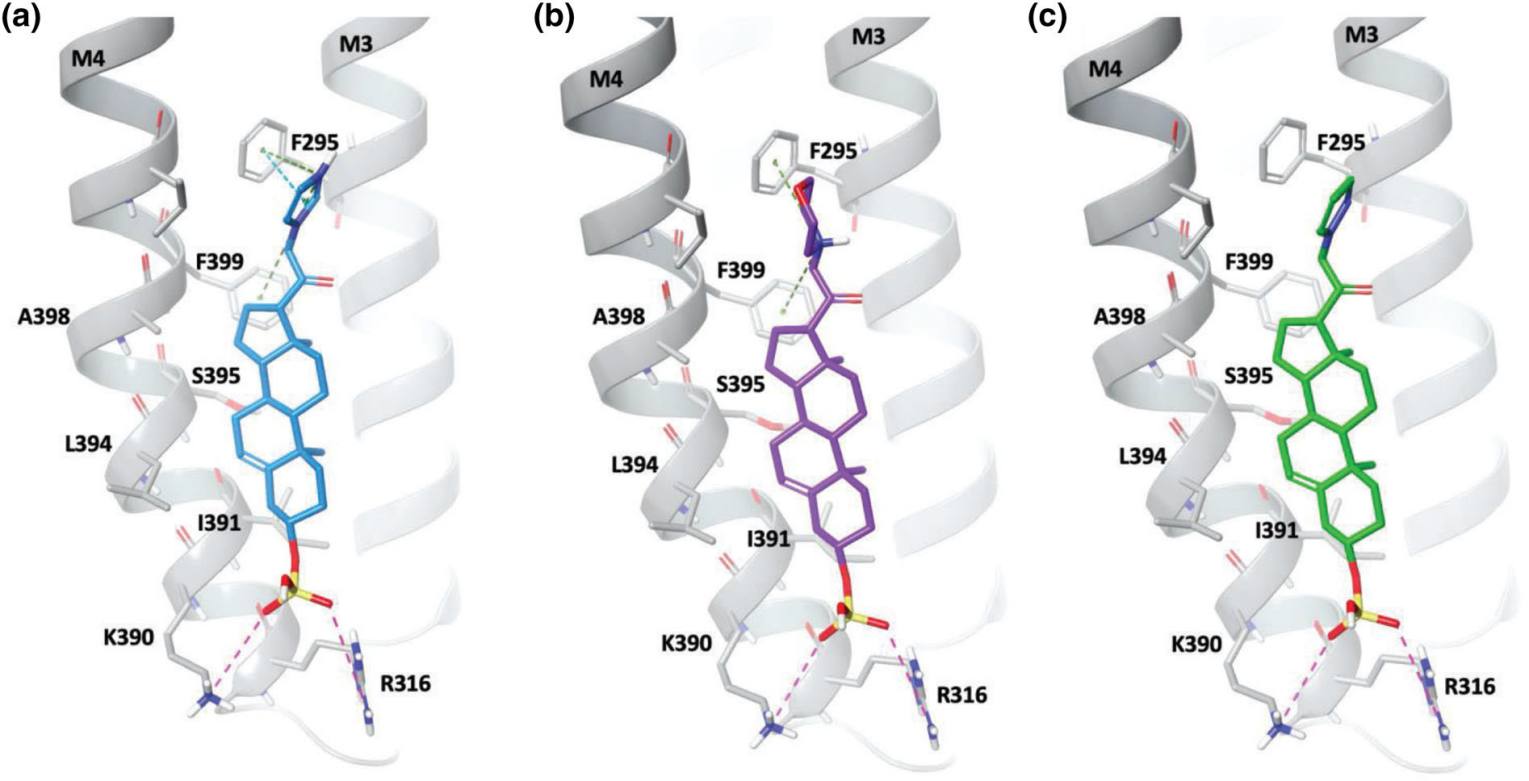
Proposed binding modes for pregnenolone sulfate analogues at the transmembrane domain (TMD) site in α1β3γ2L GABA_A_Rs. Representative binding modes for compounds **3** (a), **5** (b) and **6** (c) at the α1 subunit TMD 3 and 4 are shown. The pink dashed lines represent potential charge–charge interactions. The blue dashed lines indicate aromatic π–π interactions. The green dashed lines represent cation–π interactions. Key residues are labelled. Modelling is performed in Maestro Schrödinger and images are rendered in PyMOL (Molecular Graphics System).

Specifically, the positively charged analogues engaged in strong cation–π interactions with α1^F295^, a residue with an aromatic side chain in the TMD M3. The electrostatic pull of compounds **3** and **5** towards α1^F295^ also enabled strong aromatic face‐to‐face (π–π) interactions with F295 for compound **3** (Figure 5) and brought α1^F399^ into proximity allowing cation–π interactions with the positive charge of compound **5**. Overall, these analogue poses strengthened the binding of compounds **3** and **5** to the upper parts of the α‐helices in the TMD (five to six helical turns from the bottom of the TMD in Figure 5). By contrast, compound **6** was unable to form the same continuous cation–π interactions with F295 and/or F399, most likely due to its uncharged nature. Nevertheless, all three ligands showed the same hydrophobic contacts with the other side chains across M3 and M4 that included I391, L394 and A398 (hydrophobic box; Laverty et al., 2017) (Figure 5).

The binding site interactions of compounds **3** (cation–π and π–π) and **5** (cation–π) with α1^F295^ and α1^F399^ clearly resulted in reduced inhibitory potencies for accelerating GABA current decay compared with compound **6**. This indicated a key role for α1^F295^ and α1^F399^ in controlling the decay kinetics of the GABA_A_R in the presence of selected PS analogues. To examine their role, α1^F295^ and α1^F399^ were subject to mutagenesis and electrophysiology with compounds **5** and **6**, which exhibited the largest difference (2‐log fold) in potency for accelerating GABA current decay.

Expanding the pKa analysis to the other PS analogues revealed that compounds **3**, **4**, **5** and **7** are charged on ring D, whereas compounds **2**, **6** and **8** are uncharged. From this, it became evident that compounds **2** and **6**, as well as compounds **4** and **5**, share chemical/charge features that may be indicative of their functional characteristics (Figure 4c).

### 3.4 Substituting PS binding site residues

Following the results of the computational modelling, single and dual alanine substitutions were incorporated into the α1 subunit to investigate the importance of F295 and F399 for interacting with compounds **5** and **6**. Such a substitution eliminates the aromatic nature of these phenylalanine (Phe) residues, thereby disrupting the ability of compound **5** to engage in cation–π interactions with Phe.

Compounds **5** and **6** were applied to α1^F295A^β3γ2L, α1^F399A^β3γ2L and α1^F295A,F399A^β3γ2L and to wild‐type α1β3γ2L receptors for comparison. We first assessed the effects of F295A and F399A on the inhibitory potency of the compounds on the GABA steady‐state current.

**FIGURE 6:**
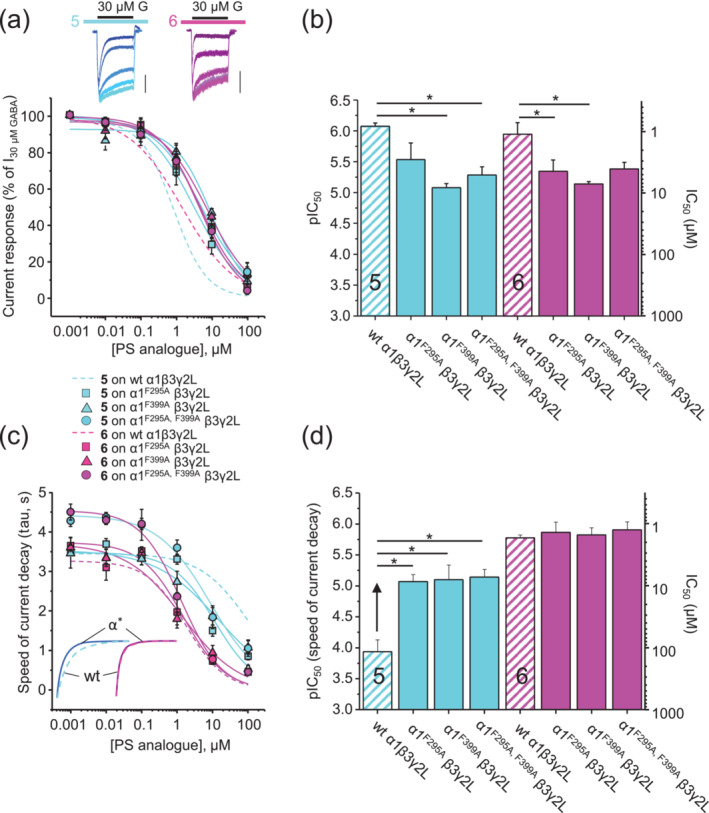
GABA‐activated currents and the effects of compounds **5** and **6** on wild‐type (wt) α1β3γ2L and mutants α1^F295A^β3γ2L, α1^F399A^β3γ2L and α1^F295A, F399A^β3γ2L GABA_A_Rs expressed in HEK cells. (a) Upper panel, overlaid representative 20 s duration of GABA (30 μM) currents for α1^F295A, F399A^β3γ2L in the presence of increasing concentrations of **5** and **6** (0.01 [lightest colours], 0.1, 1, 10 and 100 μM [darkest colours]; scale bars: 200 pA). Lower panel, mean steady‐state GABA current inhibition curves are shown for all three mutants. (b) Bar graph of the mean steady‐state potencies of pregnenolone sulfate (PS) analogues for GABA inhibition. ANOVA comparison of pIC_50_ values for wt α1β3γ2L, α1^F295A^β3γ2L, α1^F399A^β3γ2L and α1^F295A, F399A^β3γ2L receptors indicated that inhibition potencies for **5** and **6** were affected by the mutations (for **5** and **6**: *P* < 0.05); the results from Tukey's multiple‐comparison post hoc analysis are shown on the graph. (c) Relationship between PS analogue concentration and their effect on the GABA current weighted decay time constant. Inset panel, mean exponentials illustrating increased decay speeds for GABA currents of α1^F295A, F399A^β3γ2L (α*) versus wt α1β3γ2L for **5** at 10 μM (left), but lack of change in decay speed for **6** (right). (d) PS analogue potencies determined from panel (c). ANOVA for decay‐pIC_50_ values reported a significant effect of the mutations on **5**'s effect on current decay (*P* < 0.05), but not for **6** (*P* > 0.05; see Tukey's multiple‐comparison results on graph). The data points, concentration–response curves and bars are shown in cyan for compound **5** and in pink for **6**. Wild‐type curves are dashed lines, whereas mutant receptor curves are solid lines. Bar graphs show values as pIC_50_ values ± SEM (left ordinate) and mean IC_50_ values (right ordinate). Data are from five to six experiments (see Table S1 for n numbers). Statistical significance is shown as *P < 0.05.

Each mutation reduced the inhibitory potency for both compounds. Of importance, the reduction in potencies was similar for both compounds **5** and **6** (Tables 1 and S1 and Figure 6a,b). Thus, although **5** is positively charged forming cation–π interactions with the Phe residues in the TMD, neutral **6** does not, but nevertheless, the Phe mutations did not differentiate between the inhibition potencies of **5** over **6**.

By contrast, regarding the acceleration of GABA current decay, both single and double mutants unexpectedly resulted in increased potency (reduced IC_50_ values) for **5**, without changing the potency of compound **6** (Tables 1 and S1 and Figure 6c,d). Both α1^F295^ and α1^F399^ appeared to be critical for interacting with the positive charge on **5** (as cation–π interactions), although counterintuitively, their substitution with alanine increased inhibition, thereby accelerating GABA current decay kinetics. The lack of effect of these mutations on the current decay kinetics induced by compound **6** corresponds well with its lack of cation–π interactions with both α1^F295^ and α1^F399^.

To understand the differential effects of compounds **5** and **6** on GABA current decay speeds in greater detail, we examined their activity on GABA_A_Rs exhibiting different levels of macroscopic desensitisation.

### 3.5 Analysis of receptor macroscopic desensitisation

Synaptic‐type GABA_A_Rs can undergo pronounced desensitisation during persistent channel activation (Akk et al., 2001; Gielen et al., 2015). Compounds **5** and **6** affected the decay phase of GABA current responses differently, but it is important to note that this decay phase is a composite of receptor desensitisation, changes to other receptor states and PS analogue inhibition of receptor function. We therefore hypothesised that the differential effects of compounds **5** and **6** on the decay phase may reflect preferential binding of the compounds to different kinetic states of the receptor (i.e., open, closed, preactivated and/or desensitised).

**FIGURE 7:**
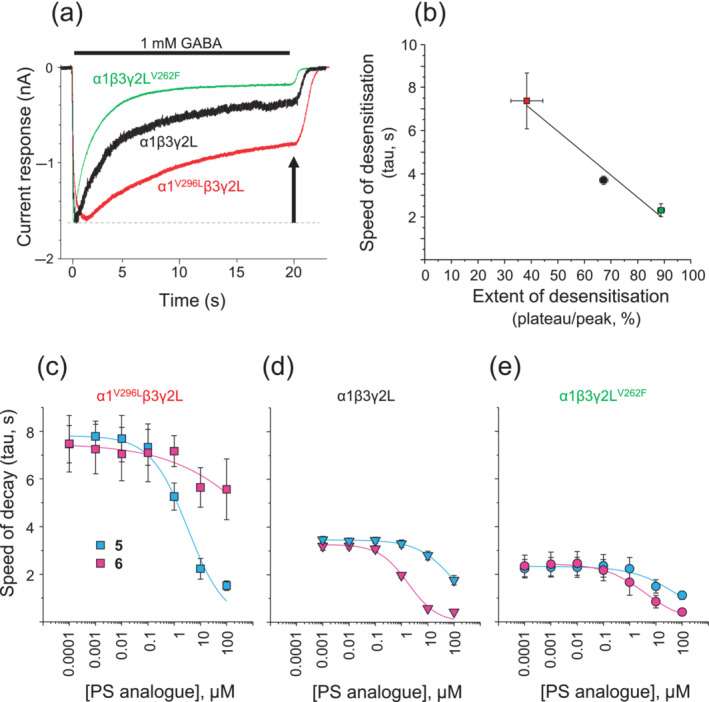
Differential modulation of GABA current decay by compounds **5** and **6** for wild‐type α1β3γ2L and two desensitisation receptor mutants: α1^V296L^β3γ2L and α1β3γ2L^V262F^. (a) Peak‐scaled overlaid GABA currents evoked by 1 mM of GABA (bar) applied to the three receptors. An arrow indicates the steady‐state current after 20 s where the extent of desensitisation was measured. (b) Relationship between the extent of desensitisation and the speed of GABA current decay (τ_w_) fitted by linear regression (y = −0.104x + 11.23) for α1β3γ2L (black), α1^V296L^β3γ2L (red) and α1β3γ2L^V262F^ (green). (c–e) Curves showing the relationship between pregnenolone sulfate (PS) analogue concentration and the effect on GABA current weighted decay time constant for compounds **5** (pink) and **6** (cyan) on the minimally desensitising α1^V296L^β3γ2L receptor (c), the wild‐type‐receptor α1β3γ2L (d) and the highly desensitising receptor α1β3γ2L^V262F^ (e). Data points are means ± SEM from five to six experiments (see Table S1 for n numbers).

To investigate, we used receptors exhibiting different degrees of desensitisation. Previously, we identified single residue mutants that affected the speed and extent of desensitisation (Gielen et al., 2015). In this study, we compared the extent of macroscopic desensitisation of wild‐type α1β3γ2L (by 68 ± 1.6%) and mutants α1^V296L^β3γ2L (39 ± 6.0%) and α1β3γ2L^V262F^ (89 ± 1.3%) receptors by measuring the steady‐state GABA current after 20 s of perfusion with 1 mM of GABA. Notably, desensitisation was markedly reduced (α1^V296L^) or increased (γ2^V262F^) compared with WT receptors (*P* < 0.05; Figure 7a).

These receptors also were compared for their GABA current decay times during the 1 mM (20 s) of GABA applications. The mean weighted decay time constants (τ_w_) varied significantly for wild‐type α1β3γ2L (3.7 ± 0.17 s), α1^V296L^β3γ2L (7.4 ± 1.3 s) and α1β3γ2L^V262F^ (2.3 ± 0.28 s; *P* < 0.05). Furthermore, we noted that the speed of decay and the extent of desensitisation were linearly related, indicating that we could use the weighted decay time constants as a proxy measure of receptor desensitisation (*R* ^2^ = 0.9738; Figure 7b).

Interestingly, whereas compound **6** affected the GABA current decay speeds more potently than **5** for wild‐type α1β3γ2L (*P* < 0.05; Table 1 and Figure 7d) and α1β3γ2L^V262F^ receptors (*P* < 0.05; Table 1 and Figure 7e), this order was reversed with the slowly desensitising receptor α1^V296L^β3γ2L, where compound **5** was significantly more potent than **6** (*P* < 0.05; Tables 1 and S1 and Figure 7c). This suggests that compound **6** preferentially inhibits receptors that incorporate a desensitised state(s), and when the probability of entry into a desensitised state is low, as for α1^V296L^β3γ2L, compound **6** is less effective at increasing the GABA current decay phase.

By contrast, compound **5** displayed an opposing profile changing from a low potency inhibitor at wild‐type α1β3γ2L to significantly higher potency at α1^V296L^β3γ2L (*P* < 0.05; Tables 1 and S1 and Figure 7c–e). As desensitisation states play a less dominant role in the kinetics of α1^V296L^β3γ2L receptors, this suggests that compound **5** has less preference for desensitised states and may prefer to inhibit other receptor states (open or closed/preactivated).

When applying compounds **5** and **6** to the fast‐desensitising α1β3γ2L^V262F^ receptor, the difference in the decay potencies for the two compounds is reduced to approximately 28‐fold with compound **6** still appearing to be more potent (Table S1 and Figure 7e). Desensitisation is exacerbated for α1β3γ2L^V262F^, and thus, we would expect compound **6** with its presumed preference for binding to desensitised states to be more potent on this isoform compared with compound **5**. Taken together, the three receptor isoforms indicate that **5**, unlike **6**, is potentially less likely to bind to the desensitised state of the receptor.

### 3.6 GABA_A_R kinetic modelling

**FIGURE 8:**
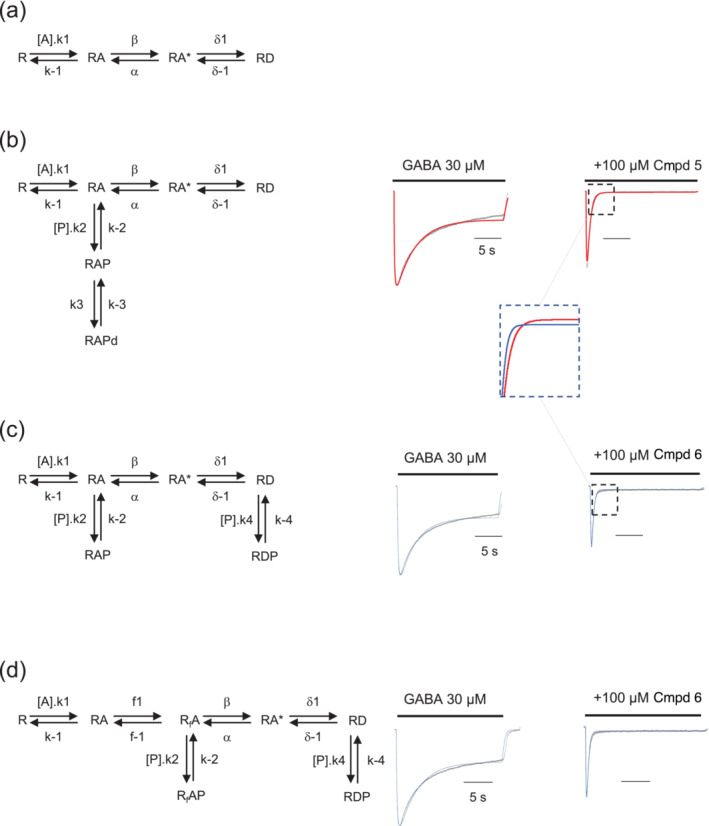
Waveform fitting of GABA currents for sulfated neurosteroids inhibited GABA_A_Rs. (a) Simplified receptor model illustrating an unbound, inactive GABA_A_ receptor (R), to which GABA_A_ binds forming the inactive RA state (that also represents here, one or more preactivated closed states). RA* is the activated receptor open channel before moving to a desensitised state RD. (b) Model for pregnenolone sulfate analogue (P) **5** that is shown binding to RA forming RAP that also proceeds to a deep blocked closed state, RAPd. (c) Model for compound (Cmpd) **6** that binds to one or more closed states, RA (forming RAP), in addition to binding to the desensitised state, RD, to form RDP. (d) Expansion of the kinetic model in panel (c) to show a preactivated state (R_f_A) bound by Cmpd **6**, instead of binding to state RA, to form R_f_AP. To the right of the kinetic models, the respective waveform fitted GABA currents are shown (red, blue) overlaid on the experimental GABA currents (black). These currents are activated by 30 μM of GABA with Cmpd **5** binding to the closed/preactivated state (RA; b) and Cmpd **6** binding to GABA‐bound closed states (RA) and to RD (c). The inset panel in (b) shows at higher resolution the faster decay induced by Cmpd **6** compared with Cmpd **5**, which for the latter also has a negligible steady‐state current. Values for the rate constants are as follows: Model (b), k1 = 9.79E8 M^−1^s^−1^; k‐1 = 1.25E4 s^−1^; β = 6.10E3 s^−1^; α = 2.28E3 s^−1^; δ1 = 2.48E‐1 s^−1^; δ‐1 = 3.90E‐2 s^−1^; k2 = 6.29E4 M^−1^s^−1^; k‐2 = 2.33E‐1 s^−1^; k3 = 9.21E5 s^−1^; k‐3 = 1.40E‐1 s^−1^; Model (c), k1 = 3.27E8 M^−1^s^−1^; k‐1 = 2.00E4 s^−1^; β = 1.44E4 s^−1^; α = 1.45E4 s^−1^; δ1 = 8.72E‐1 s^−1^; δ‐1 = 9.22E‐2 s^−1^; k4 = 2.34E5 M^−1^s^−1^; k‐4 = 1.84E2 s^−1^; k2 = 1.29E5 M^−1^s^−1^; k‐2 = 9.00E‐2 s^−1^; Model (d), k1 = 3.27E8 M^−1^s^−1^; k‐1 = 2.00E4 s^−1^; f1 = 1.03E4 s^−1^; f‐1 = 1.05E4 s^−1^; β = 1.18E4 s^−1^; α = 1.43E4 s^−1^; δ1 = 1.18E0 s^−1^; δ‐1 = 8.07E‐2 s^−1^; k2 = 1.73E5 M^−1^s^−1^; k‐2 = 1.05E‐1 s^−1^; k4 = 1.84E5 M^−1^s^−1^; k‐4 = 3.07E2 s^−1^.

The results obtained with compounds **5** and **6** were further investigated using kinetic modelling to explore plausible explanations as to why they possessed similar IC_50_ values for inhibiting GABA‐activated currents but showed different potencies for accelerating current decay, with compound **6** exhibiting the higher potency. One potential explanation involved binding of the analogues to discrete receptor activation states. To investigate, simplified GABA_A_R models were constructed, incorporating some or all the following receptor states: an unbound closed state (R); GABA‐bound closed ‘composite’ state (RA) that incorporates one or more preactivation states that are not explicitly indicated or a closed (RA) and separate preactivated (RfA) state; a GABA‐bound activated state (RA*); and a desensitised state (RD) accessed via the receptor's activated state. These simple models enable the generation of simulated GABA‐dependent currents, with values for the rate constants determined initially empirically in broad accord with published parameters and manually adjusted to provide seed values before performing waveform fitting with unconstrained rate constants (Figure 8a).

A wide range of plausible simplified models were created and systematically explored for their suitability in simulating the experimental currents of compounds **5** and **6** at various concentrations. To do so, we examined both the inclusion of new PS analogue‐bound receptor states (e.g., RAP) and variations in microscopic rate constants, with all variations being assessed in subsequent simulations. Following these kinetic model assessments, we settled on the simplest models that could best describe the observed GABA responses in the presence of compounds **5** or **6**. These models are described below.

To reproduce the block by compound **5**, a new state was required to enable the binding of this PS analogue to just a composite closed or preactivated state represented by RAP (Figure 8b). This produced a blocking phenotype that is characterised by rapid inhibition following PS binding to a closed state of the receptor (forming RAP), but this alone caused limited depression of the steady‐state current. To achieve the latter and reproduce more accurately the experimental data, RAP was permitted to traverse into a deep blocked state denoted by RAPd. This broadly recapitulated the blocking phenotype for compound **5** on α1β2γ2L receptors depressing the GABA steady‐state current in preference to the initial peak current and accelerating the current decay (Figure 8b, see inset).

However, the model adopted for compound **5** was inadequate to account for the blocking activity of compound **6**. To enable compound **6** to block with a similar potency to compound **5** (i.e., similar steady‐state IC_50_), but with faster kinetics, required binding to two receptor states, RA and also a desensitised state of the receptor, RD (Figure 8c), the latter in accord with previous experimental observations with PS (Seljeset et al., 2018). Binding to RA provided increased GABA current decay in the presence of compound **6**, and binding to the desensitised state, RD, enabled a depression of the steady‐state current again in accord with the experimental data (Figures 3e and 8c, see inset). Also notable from the waveform fitting and the rate/conformational constant values was that the PS analogues appeared to compromise both gating of the receptor and its procession into the preactivated state (see below), again indicating that the PS analogues affecting several receptor states can contribute to the increased macroscopic desensitisation of the GABA currents.

We also considered simply shifting PS binding from RD to the activated RA* as the preferred receptor state targeted by the pregnenolone analogue, which produced notable changes in GABA current profile. The receptor sensitivity to block via RA* was similar to that for compound **5** binding to RA, but the current decay was now markedly increased. However, this variation on the model is less favoured because it does not reflect our previous observations, suggesting that PS derivatives show no use dependence, nor a profile expected of an activated receptor blocker (Akk et al., 2001, 2008; Seljeset et al., 2015, 2018). Furthermore, we examined PS analogue binding to the R state, that is, with no GABA bound, but fits to the experimental data were very poor, suggesting that GABA occupancy is required even though we do not observe significant use dependence (Seljeset et al., 2018).

We next explored an expansion in the number of closed states and, in particular, a role for the preactivation state, R_f_A, as a principal binding state of the receptor for compound **6** (Figure 8d). The results obtained from waveform fitting suggested that compound **6** inhibition could also be accounted for by this analogue binding to the preactivation state together with a desensitised state. We are unable to distinguish any binding preference between the closed and preactivated states. The same conclusion, in regard to binding to different closed states, was reached with compound **5** when this analogue was permitted to bind to just a preactivated state of the receptor (not shown).

Taken together, the experimental results and simulations suggested that by chemically altering the structure of the PS derivatives, a degree of receptor state targeting may have been achieved, which, from interpretation of the model results, shifts predominantly from just one or more closed/preactivated states for compound **5** (Figure 8b) to closed/preactivated and desensitised states for compound **6** (Figure 8c,d). Of course, it must be noted that this is a simplified reductionist model that we have applied; it does not discount other more complex models from explaining the data (nor does it exclude additional blocked/closed states during the inhibition by PS), but it does account for the experimental data accrued in this study in a simplified form.

## 4 DISCUSSION

There is a need for innovation in future drug development with the aim of not only targeting specific clinically relevant receptor subtypes but also enabling modulation of functional and/or kinetic characteristics of the target macromolecule. We have, in this study, focused on a classic synaptic‐type GABA_A_R and explored the differential modulation of its kinetic states with new PS analogues.

PS is known to bind to and modulate most GABA_A_R isoforms (Seljeset et al., 2015). Although a TMD inhibitory NS binding site has been identified in the α1 subunit, from using an α1‐GLIC receptor chimera (Laverty et al., 2017), evidence suggests that additional different PS binding sites might exist in other GABA_A_R isoforms and/or subunits (Seljeset et al., 2015). Here, we have utilised structural information from the chimeric α1 subunit PS binding site, to try to understand the differentiating pharmacological profiles observed with our novel C‐21 *N*‐heterocycle‐substituted PS analogues. These different binding profiles may be useful in considering new therapeutic approaches for some channelopathies involving GABA_A_Rs. These are often associated, for example, with idiopathic epilepsies (Bernard & Shevell, 2008). Resulting seizures emanate due to abnormal GABA_A_R function and/or distribution. One of the generalised epilepsies, absence seizures, is linked to defects in tonic inhibition mediated by extrasynaptic GABA_A_Rs (Chuang & Reddy, 2020; Cope et al., 2009; Lee & Maguire, 2014; Schipper et al., 2016). These GABA_A_Rs, which are situated outside synaptic densities, are most likely composed of α4/5/6, β1/2/3 and δ subunits, depending on brain region and neuronal cell type (Glykys et al., 2008; Lee & Maguire, 2014; Olsen & Sieghart, 2009). However, it should be noted that classic synaptic GABA_A_Rs, like α1β2/3γ2, also migrate between synaptic and extrasynaptic regions as part of synaptic turnover and plasticity and may therefore contribute to tonic inhibition (Thomas et al., 2005). Indeed, we cannot discount that a component of tonic activation stems from γ‐containing GABA_A_Rs residing at inhibitory synapses.

In the context of this study, specific γ2 gene mutations (K289M and R139G) have been associated with different forms of epilepsy, and these have interestingly been linked directly to altered GABA_A_R macroscopic desensitisation (Audenaert et al., 2006; Baulac et al., 2001). It was therefore interesting that one of our PS analogues, compound **6**, seemingly displayed a preference for desensitised states of the α1β3γ2 receptor. In this study, we have focused on mutations only in the TMD, around the desensitisation gate (Gielen et al., 2015) and the proposed PS binding site(s) (Laverty et al., 2017). However, we note that other residues in the GABA_A_R that are distal to the desensitisation gate can affect macroscopic desensitisation (Klopotowski et al., 2021). Our focus on the desensitisation gate, a feature also observed in structural studies (Laverty et al., 2019), reflects its proximity to the proposed PS binding site, but regulation of desensitisation can be influenced by structures other than the desensitisation gate presumably by initiating distal conformational wave or rigid body change in the receptor. Thus, compound **6** may also bind to closed states that influence the course of macroscopic desensitisation, including binding to a preactivated state of the receptor. Although speculative and acknowledging the caveats above on conformational transmission, compound **6** being uncharged may bind and increase the hydrophobic cuff near the base of the ion channel facilitating formation of a desensitised state, a feature that compound **5** in its charged state is unable to replicate. Moreover, we do not think there are tangible changes to binding affinity with both k1 and k‐1 for GABA largely unaffected by compounds **5** and **6** and with GABA current rise times also appearing very similar.

Our hypothesis that compounds **5** and **6** are stabilising distinct desensitised state(s) of the GABA_A_R, leading to differential decay kinetics, accords with a recent paper that suggested that PS stabilised a desensitised state of the receptor, which was distinct from the ‘classic’ transmitter‐induced desensitised state (Pierce et al., 2022).

Because most neurological diseases that implicate GABA_A_Rs often involve some subtle imbalance in the fundamental relationship between excitation and inhibition, any realignment must be graded and measured. This highlights the need for new drugs that are potent but without being overly efficacious in their effects. Naturally, subtype‐selective drugs are desirable but have also been elusive when attempting to target most known GABA_A_R binding sites. To date, benzodiazepines have been the most successful drug group exhibiting subtype selectivity, but the side effects of these drugs are pronounced and often problematic (Mohler, 2015, 2002; Sigel & Ernst, 2018). One alternative way to progress could be to search for defined effects of novel drugs on the kinetics of their target receptor, which may alter and improve their overall side‐effect profiles.

The protonation states of ligands and of residues in binding sites are evidently crucial for interactions and bond formation during the ligand binding process (Chenprakhon et al., 2012; Petukh et al., 2013). Our pKa analysis highlighted a clear difference in charge associated with the PS analogues **5** and **6** by assessment in DMSO, which emulates the hydrophobic environment of the cell membrane lipid bilayer. To examine the consequences of pK_a_ variation, molecular docking and MD simulations of selected ligands suggested that such a charge differential could account for marked differences in binding modes where only positively charged analogues engaged in strong cation–π interactions with α1^F295^. Throughout the 12‐ns MD simulation, it was evident that such strong ‘interactive pull’ seems to also bring the PS ligands into proximity towards α1^F399^ allowing for further cation–π interactions with F295 and/or F399, most likely due to their lack of charge. As in silico studies showed that the examined ligands have the same hydrophobic contacts across M3 and M4, we envisioned a crucial role for either F295 or F399 in the observed differential pharmacological effects and designed in vitro mutagenesis studies accordingly. We anticipated that differential molecular mechanisms of action for compounds **3** and **5** probably resulted in a reduced inhibitory potency for accelerating GABA current decay, compared with compound **6**. Supported by a mutagenesis study around both amino acids (Figure 6), we concluded a key role for α1^F^ ^295^ and α1^F399^ in controlling the decay kinetics of the GABA_A_R in the presence of selected PS analogues, governed by the chemical nature of the PS ligands.

Taken together, this emphasises the importance of performing thorough chemical analyses of novel drugs in combination with pharmacological testing.

In summary, this new set of structurally variant PS analogues has highlighted that differential inhibitory effects can be achieved by targeting specific states of GABA_A_Rs. The chemical substitutions in compounds **5** and **6**, which resulted in their differential potencies on increasing GABA current decay without affecting steady‐state inhibition, suggest that these PS analogues affect specific receptor closed states. Our observations highlight the potential for refining and optimising known drug structures that could lead to next‐generation drugs with the ability to modulate precise kinetic characteristics of receptors and ion channels. Such an outcome could result in novel therapeutics with not only improved targeting to overactive synapses but also enhanced side‐effect profiles.

## AUTHOR CONTRIBUTIONS

Yue Xu, Jacob Krall and Bente Frølund designed the chemistry synthetic schemes, and Yue Xu performed organic chemistry synthesis and pKa analysis. Martin Mortensen designed and performed receptor mutagenesis and the electrophysiology experiments. Margot Ernst undertook structural receptor analysis. Mohamed A. Shehata performed molecular dynamics studies, analyses and supervised modelling efforts. Martin Mortensen and Trevor G. Smart developed and analysed the kinetic receptor models. Both Martin Mortensen and Yue Xu contributed equally to this study. Bente Frølund and Trevor G. Smart directed the study, wrote the manuscript and obtained funding to support the work. All authors contributed to scientific discussions, data interpretation and writing of the manuscript.

## CONFLICT OF INTEREST STATEMENT

The authors have no conflict of interest with any other party.

## DECLARATION OF TRANSPARENCY AND SCIENTIFIC RIGOUR

This Declaration acknowledges that this paper adheres to the principles for transparent reporting and scientific rigour of preclinical research as stated in the *BJP* guidelines for Design and Analysis, and as recommended by funding agencies, publishers and other organisations engaged with supporting research.

## Supporting information


**Movie S1.** Supporting Information

**Movie S2.** Supporting Information

**Movie S3.** Supporting Information

**Table S1.** Inhibitory potency for PS analogues 5 and 6 inhibiting GABA currents

**Data S1.** Supporting Information

## Data Availability

The data that support the findings of this study are available on reasonable request from the corresponding authors. Some data may not be made available because of privacy or ethical restrictions.
