# Supplementary material for: Pregnenolone sulfate analogues differentially modulate GABAA receptor closed/desensitised states
Source: Br J Pharmacol. 2023 Jun 2;180(19):2482–99. doi: 10.1111/bph.16143 (PMC10952582; doi:10.1111/bph.16143)
Supplement: Supplementary file 4 — Table S1. Inhibitory potency for PS analogues 5 and 6 inhibiting GABA currents [file BPH-180-2482-s002.pdf]

Supplementary Table 1

Parameter	$GABA_AR$ mutant	**5**	**6**
Steady-state inhibition (pIC_{50} ± SEM; IC_{50})	$\alpha1^{F295A}\beta3\gamma2L$	5.533 ± 0.272 (2.9 µM; n = 6)	5.346 ± 0.182 (4.5 µM; n = 6)
	$\alpha1^{F399A}\beta3\gamma2L$	5.078 ± 0.066 (8.4 µM; n = 6)	5.137 ± 0.042 (7.3 µM; n = 5)
	$\alpha1^{F295A,\ F399A}\beta3\gamma2L$	5.282 ± 0.138 (5.2 µM; n = 6)	5.382 ± 0.107 (4.2 µM; n = 6)
	$\alpha1^{V296L}\beta3\gamma2L$	4.685 ± 0.473 (21 µM; n = 5)	n.m. (>100 µM; n = 5)
	$\alpha1\beta3\gamma2L^{V262F}$	4.849 ± 0.186 (14 µM; n = 5)	4.60 ± 0.161 (25 µM; n = 5)
Decay inhibition (pIC_{50} ± SEM; IC_{50})	$\alpha1^{F295A}\beta3\gamma2L$	5.072 ± 0.113 (8.5 µM; n = 6)	5.870 ± 0.167 (1.4 µM; n = 6)
	$\alpha1^{F399A}\beta3\gamma2L$	5.103 ± 0.239 (7.9 µM; n = 6)	5.828 ± 0.118 (1.5 µM; n = 6)
	$\alpha1^{F295A,\ F399A}\beta3\gamma2L$	5.142 ± 0.128 (7.2 µM; n = 6)	5.910 ± 0.130 (1.2 µM; n = 6)
	$\alpha1^{V296L}\beta3\gamma2L$	5.490 ± 0.052 (3.2 µM; n = 5)	n.m. (>100 µM; n = 6)
	$\alpha1\beta3\gamma2L^{V262F}$	4.150 ± 0.080 (71 µM; n = 5)	5.540 ± 0.174 (2.9 µM; n = 5)

Supplementary Table 1. Inhibitory potency for PS analogues compounds **5** and **6** inhibiting GABA steady-state currents and increasing the speed of GABA current decay. These are represented by pIC_{50} and IC_{50} values for α1β3γ2L $GABA_ARs$ expressed in HEK293 cells. Mean IC_{50} values are calculated from pIC_{50} values. Where inhibition curves did not contain complete information for sigmoidal curve fits, IC_{50} values were estimated by extrapolation or designated as ‘not measureable’ (n.m.). In these cases predictions were made that the IC_{50} values were likely to be higher (>) than 100 µM. The pIC_{50} values are mean ± SEM where n is the number of experiments.
