# Supplementary material for: Pregnenolone sulfate analogues differentially modulate GABAA receptor closed/desensitised states
Source: Br J Pharmacol. 2023 Jun 2;180(19):2482–99. doi: 10.1111/bph.16143 (PMC10952582; doi:10.1111/bph.16143)
Supplement: Supplementary file 5 — Data S1. Supporting Information [file BPH-180-2482-s003.pdf]

SUPPORTING INFORMATION

Synthetic chemistry:

General procedure: Compound **10-14** were synthesized in a general procedure exemplified by compound **11**: To a solution of compound **9** (100 mg, 0.25 mmol, 1 eq) and piperidine (25 mg, 0.29 mmol, 1.1 eq) in 5 ml acetone was added K_2CO_3 (174 mg, 1.26 mmol, 5 eq) and KI (21 mg, 0.17 mmol, 0.5 eq). The resulting mixture was refluxed for 6 h, followed by evaporation of solvent. The residue was dissolved in 5ml water and 8ml ethyl acetate and the aqueous phase was extracted by ethyl acetate (2 x 5 ml). The combined organic phase was evaporated and re-dissolved in 20 ml H_2O / Acetonitrile = 1:1 and purified by prep-HPLC to yield compound **11** as an off white solid (60 mg, 0.15 mmol, 59 %)

General procedure for sulfate (A): Compound **2**, **6** and **8** were synthesized in a general procedure exemplified by compound **2**: To a solution of compound **9** (47 mg, 0.12 mmol, 1 equiv) in 8.5 ml freshly dried chloroform was added pyridine sulfur trioxide (38 mg, 0.24 mmol, 2 equiv). The reaction was stirred at room temperature and monitored by analytic-HPLC to ensure that starting material **9** was all reacted. The resulting mixture was then allowed to stand at -5°C for 2 hours and then quickly filtered. The filtrate was evaporated and dried in vacuo over 2 days to afford the corresponding sulfate **2** as an off-white solid (34 mg, 0.06 mmol, 52 %).

General procedure for sulfate (B): Compound **3-5** and **7** were synthesized in a general procedure exemplified by compound **3**: To a solution of compound **10** (24 mg, 0.06 mmol, 1 equiv) in 3 ml DMF was added pyridine sulfur trioxide (11 mg, 0.07 mmol, 1.1 equiv). The reaction was heated at 60°C along with deposit formation and monitored by analytic-HPLC to ensure that starting material **10** was all reacted. After cooling to room temperature, the resulting precipitate was filtered and washed with ethyl acetate repeatedly. The solid was dried in vacuo overnight to yield compound **3** as an off-white solid (17 mg, 0.03 mmol, 58 %).

Pregn-5-en-21-bromo-20-one-3β-sulfate pyridinium (2). HR-MS (ESI): m/z 475.0963 (M-1) calcd for $C_{21}H_{30}BrO_5S$. ^{1}H NMR (400 MHz, $CDCl_3$): δ 8.92 (dd, J_1 = 6.6 Hz, J_2 = 1.4 Hz, 2H), 8.47-8.42 (m, 1H), 7.95 (dd, J_1 = 7.8 Hz, J_2 = 6.5 Hz, 2H), 5.41-5.37 (m, 1H), 3.95-3.87 (m, J_1 = 16.0 Hz, J_2 = 12.0 Hz, 2H), 3.57-3.48 (m, 1H), 2.82 (t, J = 9.0 Hz, 1H), 2.68-0.93 (m, 19H), 1.01(s, 3H), 0.66(s, 3H). ^{13}C NMR (151MHz, $CDCl_3$): δ 202.1, 145.4, 142.4, 140.1, 127.0, 122.0, 78.8, 60.4, 56.7, 49.7, 44.8, 39.1, 38.6, 37.1, 36.4, 36.2, 31.8, 31.7, 28.7, 24.5, 23.6, 21.0, 19.3, 13.5.

3β-Sulfate-pregn-5-en-21-(imidazol-1'-yl)-20-one (3). LC-MS (ESI): t_R = 1.71 min, m/z = 461.6 (M-1). ^{1}H NMR (600 MHz, DMSO-d_6): δ 8.22 (s, 1H), 7.28 (d, J = 43.0 Hz, 2H), 5.31-5.27 (m, 1H), 5.22-5.03 (m, 2H), 3.88-3.81 (m, 1H), 2.72-0.97 (m, 20H), 0.96(s, 3H), 0.61(s, 3H). ^{13}C NMR (151 MHz, DMSO-d_6): δ 203.6, 140.7, 138.2, 123.2, 121.3, 120.8, 75.1, 59.4, 56.0, 49.3, 43.9, 39.3, 37.4, 36.9, 36.1, 34.3, 31.3, 31.2, 28.9, 28.7, 24.0, 22.5, 19.0, 13.0.

3β-Sulfate-pregn-5-en-21-(piperidin-1'-yl)-20-one (4). Yield: 19 mg (50 %) as an off-white solid. LC-MS (ESI): t_R = 1.78 min, m/z = 478.7 (M-1). ^{1}H NMR (600 MHz, DMSO-d_6): δ 9.46 (brs, 1H), 5.33-5.26 (m, 1H), 4.38-4.10 (m, 2H), 3.41-3.26 (m, 2H), 3.01-2.92 (m, 2H), 2.64-2.59 (m, 1H), 2.18-0.97(m, 26H) 0.96 (s, 3H), 0.61 (s, 3H). ^{13}C NMR (151 MHz, DMSO-d_6): δ 205.3, 140.9, 121.1, 76.2, 64.3, 60.1, 56.4, 53.7, 49.7, 44.7, 39.1, 37.8, 37.2, 36.5, 31.7, 31.6, 29.0, 24.4, 22.7, 22.4, 21.5 20.8, 19.4, 13.4.

3β-Sulfate-pregn-5-en-21-(morpholin-2'-yl)-20-one (5). Yield: 11 mg (34 %) as an off-white solid. LC-MS (ESI): t_R = 1.65 min, m/z = 480.7 (M-1), m/z = 482.7 (M+1). ^{1}H NMR (600 MHz, DMSO-d_6): δ 10.14 (brs, 1H), 5.32-5.27 (m, 1H), 4.47-4.20 (m, 2H), 3.98-3.88 (m, 2H), 3.87-3.81 (m, 1H), 3.79-3.71 (m, 2H), 3.42-3.33 (m, 2H), 3.21-3.10 (m, 2H), 2.65-2.59 (m, 1H), 2.19-0.97 (m, 19H), 0.96 (s, 3H), 0.61 (s, 3H). ^{13}C NMR (151 MHz, DMSO-d_6): δ 208.2, 141.4, 121.3, 75.5,

64.3, 63.4, 60.1, 56.4, 52.4, 49.7, 44.7, 42.5, 37.8, 37.3, 36.5, 31.8, 31.6, 29.2, 24.4, 22.5, 20.9, 19.4, 13.4.

3β-Sulfate-pregn-5-en-21-(pyrazol-1'-yl)-20-one (6). Yield: 17 mg (34 %) as an off-white solid. LC-MS (ESI): t_R = 2.19 min, m/z = 461.6 (M-1). ^{1}H NMR (400 MHz, DMSO-d_6): δ 7.65 (d, *J* = 2.1 Hz, 1H), 7.44 (d, *J* = 1.7 Hz, 1H), 6.26 (t, *J* = 2.1 Hz, 1H), 5.31-5.26 (m, 1H), 5.16-4.97 (m, 2H), 3.88-3.79 (m, 1H), 2.66 (t, *J* = 8.21 Hz, 1H), 2.41-0.96 (m, 19H), 0.96 (s, 3H), 0.60 (s, 3H). ^{13}C NMR (101 MHz, DMSO-d_6): δ 204.7, 141.4, 139.0, 131.5, 120.4, 105.3, 69.9, 60.8, 59.2, 56.1, 49.4, 43.9, 42.1, 37.7, 36.9, 36.1, 31.4, 31.2, 28.8, 24.1, 22.4, 20.5, 19.1, 13.1.

3β-Sulfate-pregn-5-en-21-(benzimidazol-1'-yl)-20-one (7). Yield: 21 mg (53 %) as an off-white solid. LC-MS (ESI): t_R = 1.96 min, m/z = 511.6 (M-1). ^{1}H NMR (600 MHz, DMSO-d_6): δ 9.14 (brs, 1H), 7.84-7.81 (m, 1H), 7.71-7.61 (m, 1H), 7.52-7.45 (m, 2H), 5.62-5.37 (m, 2H), 5.33-5.30 (m, 1H), 3.89-3.82 (m, 1H), 2.87 (t, *J* = 9.27 Hz, 1H), 2.24-0.94 (m, 19H), 0.98 (s, 3H), 0.67 (s, 3H). ^{13}C NMR (151 MHz, DMSO-d_6): δ 203.2, 143.3, 140.8, 132.2, 125.1, 125.0, 120.8, 116.3, 112.3, 75.1, 59.4, 56.1, 55.2, 49.3, 44.0, 40.0, 37.5, 36.9, 36.1, 31.4, 31.2, 28.8, 24.0, 22.6, 20.5, 19.0, 13.0.

Pregn-5-en-21-(pyrrol-1'-yl)-20-one-3β-sulfate pyridinium (8). Yield: 96 mg (48 %) as a pink solid. LC-MS (ESI): t_R = 2.42 min, m/z = 460.7 (M-1). ^{1}H NMR (400 MHz, $CDCl_3$): δ 8.91 (d, *J* = 5.5 Hz, 2H), 8.47 (t, *J* = 7.7 Hz, 1H), 7.97 (t, *J* = 6.7 Hz, 2H), 6.58-6.56 (m, 2H), 6.21-6.19 (m, 2H), 5.41-5.37 (m, 1H), 4.68-4.53 (m, 2H), 4.43-4.33 (m, 1H), 2.69-1.08 (m, 20H), 1.01 (s, 3H), 0.68 (s, 3H). ^{13}C NMR (101 MHz, $CDCl_3$): δ 205.2, 145.4, 141.6, 140.2, 125.7, 122.0, 121.6, 109.0, 78.8, 59.7, 59.6, 56.9, 49.8, 44.7, 39.2, 39.0, 37.2, 36.5, 31.9, 31.8, 28.8, 24.6, 23.4, 21.1, 19.3, 13.6.

3β-Hydroxy-pregn-5-en-21-bromo-20-one (9). To a solution of pregnenolone (1 g, 3.16 mmol, 1 equiv) in 20 ml methanol was added pyridine (375 mg, 4.8 mmol, 1.5 equiv). The solution was stirred at reflux for 10 min before adding $CuBr_2$ (2.1 g, 9.48 mmol, 3 equiv) into it. The resulting mixture was refluxed for 1 h and monitored by HPLC. When the reaction was finished, the mixture was diluted with ethyl acetate and 1 M HCl. The aqueous phase was extracted three times and the combined organic phase was evaporated. The crude was purified by silica-chromatography (EA : Hep=1:1) to yield **9** as an off-white solid (52 %, 650 mg). HR-MS (ESI): m/z 377.1477 (M-18) calcd for C21H30BrO. ^{1}H NMR (400 MHz, $CDCl_3$): δ 5.37-5.33 (m, 1H), 3.95-3.87 (m, 2H), 3.57-3.48 (m, 1H), 2.83 (t, *J* = 12.0 Hz, 1H), 2.35-0.93 (m, 19H), 1.01(s, 3H), 0.66(s, 3H). ^{13}C NMR (151 MHz, DMSO-d_6): δ 202.0, 140.7, 121.2, 71.6, 60.3, 56.8, 49.9, 44.8, 42.2, 38.7, 37.2, 36.5, 35.8, 31.9, 31.7, 31.5, 25.5, 23.7, 21.0, 19.3, 13.4.

3β-Hydroxy-pregn-5-en-21-(imidazole-1'-yl)-20-one (10). Yield: 24 mg (61 %) as an off-white solid. ^{1}H NMR (600 MHz, DMSO-d_6): δ 9.00 (s, 1H), 7.64 (d, *J* = 25.0 Hz, 2H), 5.38-5.18 (m, 2H), 5.30-5.26 (m, 1H), 3.31-3.20 (m, 1H), 2.47 (t, *J* = 12 Hz, 1H), 2.20-0.92 (m, 19H), 0.96(s, 3H), 0.62(s, 3H). ^{13}C NMR (151 MHz, DMSO-d_6): δ 202.5, 141.3, 136.5, 123.3, 120.2, 119.4, 69.9, 59.4, 57.8, 56.0, 49.3, 44.0, 42.1, 37.3, 36.9, 36.1, 31.4, 31.3, 31.2, 24.0, 22.5, 20.5, 19.1, 12.9.

3β-Hydroxy-pregn-5-en-21-(piperidin-1'-yl)-20-one (11). Yield: 30 mg (66 %) as an off-white solid. ^{1}H NMR (600 MHz, $CDCl_3$): δ 5.36-5.32 (m, 1H), 4.00-3.92 (m, 2H), 3.65-3.54 (m, 2H), 3.55-3.49 (m, 1H), 3.15-3.03 (m, 2H), 2.47 (t, *J* = 8.8 Hz, 1H), 2.33-0.95 (m, 25H), 1.00(s, 3H), 0.65(s, 3H). ^{13}C NMR (151 MHz, DMSO-d_6): δ 201.9, 140.8, 121.2, 71.7, 63.6, 61.1, 57.0, 53.3, 49.8, 45.1, 42.2, 38.7, 37.2, 36.5, 31.8, 31.7, 31.6, 24.4, 23.0, 22.9, 21.8, 21.0, 19.4, 13.3.

3β-Hydroxy-pregn-5-en-21-(morpholin-2'-yl)-20-one (12). Yield: 27 mg (72 %) as an off-white solid. ^{1}H NMR (600 MHz, $CDCl_3$): δ 5.35-5.32 (m, 1H), 3.97 (t, *J* = 6.0 Hz, 4H), 3.87-3.78 (m, 2H), 3.55-3.50 (m, 1H), 3.35-3.22 (m, 4H), 2.51-0.95 (m, 20H), 1.00(s, 3H), 0.66(s, 3H). ^{13}C NMR (151 MHz, DMSO-d_6): δ 202.3, 140.8, 121.2, 71.7, 64.4, 64.2, 61.1, 57.0, 51.9, 49.8, 45.0, 42.2, 38.7, 37.2, 36.5, 31.8, 31.7, 31.6, 24.5, 23.0, 21.0, 19.4, 13.3.

3β-Hydroxy-pregn-5-en-21-(pyrazol-1'-yl)-20-one (13). Yield: 41 mg (61 %) as an off-white solid. ^{1}H NMR (600 MHz, DMSO-d_6): δ 7.64 (d, *J* = 6.0 Hz, 1H), 7.43 (d, *J* = 3.0 Hz, 1H), 6.26 (t, *J* = 3.0 Hz, 1H), 5.29-5.25 (m, 1H), 5.15-4.96 (m, 2H), 3.31-3.22 (m, 1H), 2.66 (t, *J* = 9.1Hz, 1H), 2.69-0.91 (m, 19H), 0.96(s, 3H), 0.62(s, 3H). ^{13}C NMR (151 MHz, DMSO-d_6): δ 204.5, 141.2, 138.8, 131.4, 120.2, 105.2, 69.9, 60.8, 59.2, 56.1, 49.4, 43.9, 42.1, 40.2, 37.7, 36.9, 36.1, 31.4, 31.2, 24.1, 22.4, 20.5, 19.1, 13.1.

3β-Hydroxy-pregn-5-en-21-(benzimidazol-1'-yl)-20-one (14). Yield: 33 mg (44 %) as an off-white solid. ^{1}H NMR (600 MHz, DMSO-d_6): δ 8.99 (s, 1H), 7.82-7.79 (m, 1H), 7.65-7.62 (m, 1H), 7.50-7.44 (m, 2H), 5.60-5.35 (m, 2H), 5.30-5.27 (m, 1H), 3.31-3.24 (m, 1H), 2.86 (t, *J* = 9.1Hz, 1H), 2.89-0.94 (m, 19H), 0.97(s, 3H), 0.66(s, 3H). ^{13}C NMR (151 MHz, DMSO-d_6): δ 203.30, 143.54, 141.30, 132.52, 124.72, 124.52, 120.24, 116.63, 112.14, 69.98, 59.45, 56.16, 55.07, 49.43, 44.03, 42.20, 37.64, 36.95, 36.13, 31.44, 31.40, 31.22, 24.09, 22.62, 20.62, 19.14, 13.08.

3β-Hydroxy-pregn-5-en-21-amino-20-one hydrochloride (15). To a solution of **2** (650 mg, 1.65 mmol, 1 equiv) in 10 ml chloroform was added hexamethlyenetetramine (230 mg, 1.65 mmol, 1 equiv) in portions. The resulting solution was allowed to stir at room temperature overnight. The precipitation was filtered and wash by chloroform repeatedly. The obtained

hexamethlyenetetrammonium salt was dissolved in 1 ml concentrated hydrochloric acid in 8ml ethanol. The mixture was stirred at room temperature for 4 days. The filtrate was evaporated and the residue was recrystallized to give **15** (350 mg, 57 %) as a white solid. ^{1}H NMR (600 MHz, DMSO-d_6): δ 5.29-5.25 (m, 1H), 4.60 (d, *J* = 4.6 Hz, 1H), 3.93-3.70 (m, 2H) 3.29-3.22 (m, 1H), 2.65 (t, *J* = 9.1 Hz, 1H), 2.18-0.95 (m, 21H), 0.94 (s, 3H), 0.59 (s, 3H). ^{13}C NMR (151 MHz, DMSO-d_6): δ 204.15, 141.29, 120.18, 69.94, 59.33, 56.08, 56.00, 49.40, 48.02, 44.07, 42.17, 37.42, 36.91, 36.09, 31.38, 31.20, 24.03, 22.31, 20.49, 19.12, 12.92.

3β-Hydroxy-pregn-5-en-21-(pyrrol-1'-yl)-20-one (16). To a solution of **15** (350 mg, 0.95 mmol) in 10 ml water was added 147 mg sodium acetate and 1 ml glacial acetic acid. The suspension was heated to 100 ℃, when 140 mg 2, 5-dimethoxytetrahydrofuran was added. After stirring at this temperature for 15 min, the mixture was cooled down and extracted with ethyl acetate. The organic phase was wash with saturated $NaHCO_3$ and brine, then dried, and evaporated. The crude was purified by silica-chromatography (EtOAc : Hep = 1:1) to give **16** (168 mg, 46 %) as an off-white solid. ^{1}H NMR (400 MHz, $CDCl_3$): δ 6.58-6.56 (m, 2H), 6.22-6.19 (m, 2H), 5.36-5.33 (m, 1H), 4.68-4.53 (m, 2H), 3.58-3.49 (m, 1H), 2.51 (t, *J* = 8.5 Hz, 1H), 2.34-0.96 (m, 19H), 1.02 (s, 3H), 0.69 (s, 3H). ^{13}C NMR (101 MHz, $CDCl_3$): δ 205.20, 140.80. 121.66, 121.36, 109.05, 71.73, 59.74, 59.60, 56.99, 49.96, 44.78, 42.29, 39.05, 37.32, 36.58, 31.94, 31.80, 31.67, 24.67, 23.48, 21.17, 19.44, 13.66.
